# Predictors of program interest in a digital health pilot study for heart health

**DOI:** 10.1371/journal.pdig.0000303

**Published:** 2023-07-31

**Authors:** Kimberly G. Lockwood, Viveka Pitter, Priya R. Kulkarni, Sarah A. Graham, Lisa A. Auster-Gussman, OraLee H. Branch

**Affiliations:** 1 Clinical Research, Lark Health, Mountain View, California, United States of America; 2 Data Science, Lark Health, Mountain View, California, United States of America; 3 Digital Health Innovations, Roche Information Solutions, Santa Clara, California, United States of America; Iran University of Medical Sciences, IRAN (ISLAMIC REPUBLIC OF)

## Abstract

Digital health programs can play a key role in supporting lifestyle changes to prevent and reduce cardiovascular disease (CVD) risk. A key concern for new programs is understanding *who* is interested in participating. Thus, the primary objective of this study was to utilize electronic health records (EHR) ***to predict interest*** in a digital health app called Lark Heart Health. Because prior studies indicate that males are less likely to utilize prevention-focused digital health programs, secondary analyses assessed sex differences in recruitment and enrollment. Data were drawn from an ongoing pilot study of the Heart Health program, which provides digital health behavior coaching and surveys for CVD prevention. EHR data were used to predict whether potential program participants who received a study recruitment email showed interest in the program by “clicking through” on the email to learn more. Primary objective analyses used backward elimination regression and eXtreme Gradient Boost modeling. Recruitment emails were sent to 8,649 patients with available EHR data; 1,092 showed interest (i.e., clicked through) and 345 chose to participate in the study. EHR variables that predicted higher odds of showing interest were higher body mass index (BMI), fewer elevated lab values, lower HbA1c, non-smoking status, and identifying as White. Secondary objective analyses showed that, males and females showed similar program interest and were equally represented throughout recruitment and enrollment. In summary, BMI, elevated lab values, HbA1c, smoking status, and race emerged as key predictors of program interest; conversely, sex, age, CVD history, history of chronic health issues, and medication use did not predict program interest. We also found no sex differences in the recruitment and enrollment process for this program. These insights can aid in refining digital health tools to best serve those interested, as well as highlight groups who may benefit from behavioral intervention tools promoted by additional recruitment efforts tailored to their interest.

## Author summary

In this study, we used electronic health record data to explore whether we could predict interest in a heart health digital lifestyle change program among potential program participants who received a study recruitment email. Potential participants were *more likely* to show program interest if they had higher body mass index, fewer elevated lab values, lower glycated hemoglobin (HbA1c), non-smoking status, and identified as White. In contrast, potential participants were equally likely to show interest regardless of sex, age, cardiovascular disease history, history of chronic health issues, and medication use. Additionally, males and females showed similar program interest and were equally represented throughout recruitment and enrollment, indicating higher than average representation of males in the Heart Health clinical study compared to most prevention-oriented digital health programs. Taken together, these insights can aid in refining digital health programs to best serve those who are interested in participating, as well as help to highlight groups of individuals who may benefit from such programs but need additional recruitment efforts or tailoring of the program to increase their interest.

## Introduction

Cardiovascular disease (CVD) is the most common health condition in the United States, with 126.9 million adults (49.2% of adults ≥20 years) living with CVD [1]. As the leading cause of death in the US and other developed nations, CVD poses substantial public health and economic burdens [1,2] making prevention and risk reduction top priorities. Importantly, CVD can be prevented or significantly reduced by maintaining a healthy lifestyle [3,4]. Behavioral interventions targeting healthy lifestyle behaviors play a critical role in preventing and managing CVD risk, as recognized by the US Preventive Services Task Force [5–7]. Traditionally, these interventions have been delivered in person [6] or via telephone [8]. However, with the rise of technology and smartphone usage, digital health programs have emerged as a convenient and accessible alternative for managing cardiovascular health [9].

Digital health programs encompass various modalities, such as mobile apps, web-based programs, text messaging, and wearable devices for remote patient monitoring. Such programs can also be integrated with standard clinical care for disease prevention and management [10]. Digital health programs offer several advantages over in-person or telephone-based programs, including increased accessibility, remote monitoring capabilities, continuous support for daily lifestyle changes, and highly personalized care [10]. These programs also have the potential to be more cost effective than traditional methods, due to reduced staffing requirements [11].

Understanding the factors that influence interest in digital health programs is vital for program design and implementation. Factors like sex, age, and health history have all been proposed as predictors of participation in digital health programs [9,12]. However, the existing evidence on predictors of interest in digital health programs is limited, as noted in recent reviews [13]. For instance, a study examining predictors of non-participation in a cardiac telerehabilitation program found that older age, lower educational level, current smoking, lower exercise capacity, and history of cardiac surgery were all associated with lower likelihood of participation [12]. Drawing on research with older adults, age is considered to have a major impact on interest in digital health lifestyle programs, with some studies indicating that older adults are less likely to take part in digital health programs, due to lower familiarity with emerging technologies [14]. However, recent research on older adults and digital health challenges this assumption [13,15,16], suggesting that conventional wisdom related to interest in digital health programs may be inaccurate. Taken together, this literature highlights the need to expand the literature on predictors of digital health program interest.

Understanding predictors of digital health program interest becomes even more critical when considering disease-specific digital health offerings. For CVD-specific digital health tools, there is a lack of published evidence testing key predictors of program interest that are especially important for CVD. For instance, there are well-documented sex differences in CVD, such that incidence of cardiac events tends to occur at an earlier age in males compared to females [4,17] and CVD prevalence is higher among males than females [1]. Moreover, males also tend to engage in more health risk behaviors and less healthcare utilization than females [18]. These sex differences underscore the importance of examining sex as a predictor of predictor of interest in digital health programs for CVD prevention. However, there is growing evidence of sex differences in utilization of prevention-focused digital health solutions. For example, males are less likely to use health apps for prevention [19], to engage with wellbeing apps or apps designed to improve health [20], to use nutrition and self-care apps [21], to engage in e-health behaviors [22], and to be aware of and use internet-based personal health records [23]. Taken together, additional research is needed focused on examining interest in CVD-specific digital health tools and whether sex differences are a key predictor.

To address these gaps in the literature, the present study aims to investigate interest in a CVD-specific digital health tool called Lark Heart Health. Heart Health is an artificial-intelligence (AI)-powered lifestyle change program that provides health behavior coaching and surveys to prevent and manage atherosclerotic cardiovascular disease (ASCVD) and coronary artery disease (CAD) by targeting key CVD risk factors. The Heart Health program is designed for primary prevention in individuals without a history of CVD or secondary prevention for those in stable condition after a cardiovascular event. The Heart Health program delivers unlimited, real-time heart health coaching that focuses on digital nutrition therapy, medication adherence counseling, and personalized guidance on weight loss, physical activity, tobacco cessation, stress, and sleep. Heart Health is fully digital and offered to adult members of Lark’s healthcare partners via a smartphone connected to the internet. The content of Heart Health is designed in accordance with guidelines from the American Heart Association, American College of Cardiology, and the National Heart, Lung, and Blood Institute. Nutrition, physical activity, and sleep recommendations in the Heart Health program are also informed by the American Diabetes Association, American Food and Drug Administration, and 2020–2025 Dietary Guidelines for Americans. Full detail on Lark’s other lifestyle change programs for diabetes prevention and hypertension care can be found in recent publications [24,25].

As described, a key concern for new programs is understanding *who* is interested in participating. Thus, a primary goal of this retrospective study was to utilize electronic health record (EHR) data ***to predict interest*** in a CVD-specific digital health app, Lark Heart Health, a program focused on CVD prevention through behavioral coaching and lifestyle change.

Primary Objective: to explore if EHR data readily available to healthcare providers (e.g., demographics, health history, health behaviors) could be used to predict a proxy measure of program interest known as “*click through”* (i.e., whether a potential participant clicked through on study recruitment materials sent via email).

Secondary Objective 1: to examine sex differences at key points in the study recruitment and initiation process for the Lark Heart Health pilot study, as measured by percentage of participants who opened the recruitment email, clicked on the personalized link in the recruitment email (i.e., clicked through), passed the prescreener, and initiated the Heart Health program. The goal was to assess whether males may be more difficult to recruit and enroll for prevention-focused digital health programs compared to females.

Secondary Objective 2: to examine sex differences in baseline characteristics among participants who initiated the Heart Health program to determine whether there were notable differences between males and females who opted to enroll in the program.

## Materials and methods

### Study setup

Data for these analyses were drawn from a real-world, non-interventional, single-arm, observational pilot study of a digital health app-based program called Lark Heart Health, which provides low-risk health behavior surveys and coaching. The study is 3-months (90 days) in duration for each participant. This pilot focuses on feasibility of deploying screener surveys and user acceptability of coaching to improve knowledge and self-management of ASCVD risk. This acceptability and feasibility pilot study received approval from Advarra Institutional Review Board (protocol Pro00061694). Appropriate safeguards were taken to prevent any unauthorized use or disclosure of personal health information and to implement the administrative, physical, and technical safeguards to protect the confidentiality, integrity, and availability of protected health information. Lark is compliant with HIPAA Privacy and Security rules and all applicable regulations. Additionally, Lark is SOC2 and HITRUST certified.

Data collection for the pilot study of the Heart Health program is ongoing, and we will report on the full results upon conclusion of the study. The present analyses focus on data provided by prospective participants during the prescreening process or shortly after program initiation, as well as deidentified EHR data from prospective participants. The data presented here include participants who initiated the study between March 31 and August 24, 2022, using Lark app version 5.2.6.

### Patient selection

#### Initial patient pool

Prior to launching the Heart Health program, a Lark healthcare partner provided Lark with a limited set of EHR data for potential program participants. To be eligible for inclusion in the EHR dataset, potential participants had to fit the following basic eligibility parameters: 40–75 years of age, English speaking, contact information available in EHR (i.e., email or phone number), and no record of a cardiovascular event/surgery or major comorbidity for at least six months prior to March 10, 2022. Cardiovascular events and surgery included stroke, heart failure, heart attack, hypertensive crisis, heart surgery, and aortic aneurysm dissection. Major comorbidities included stage 5 chronic kidney disease, acute kidney failure, and current pregnancy.

#### Recruitment process and eligibility for pilot study

The research team sent potentially eligible study participants marketing emails and/or SMS messages, and printed mailers. Recruitment emails provided a brief description of the program, describing the program as a way to improve heart health using digital health coaching that encouraged eating healthier and getting more active. Marketing materials stated that eligible participants would be provided with the Heart Health app, a digital smart scale, and opportunities for gift card incentives and a Fitbit by meeting specific engagement milestones. These recruitment materials did not provide specific details on what the study involved or the amount of possible monetary incentives. Individuals interested in participating in the study could follow a personalized link in the marketing materials to find out more information and complete the study prescreening process. All individuals who received the marketing materials had insurance coverage.

In order to confirm eligibility for the study, interested participants completed a prescreener that confirmed their eligibility and assessed the full study exclusion criteria, including: body mass index (BMI) < 25 and ≥ 50; critically serious uncontrolled health conditions that had been active in the last six months; plans to become pregnant within the next six months; recent history of a medical professional telling them not to participate in a healthy lifestyle program; a medical reason preventing them from doing 10 minutes of moderate physical exercise; and not having a smartphone with an internet connection. Because the Heart Health program focuses heavily on improving health behaviors (e.g., diet and exercise), the study also excluded individuals who reported engaging in strenuous physical activity in their leisure time and individuals who reported only healthy dietary behaviors (described in further detail in the Participant Characteristics Measures section). All participants provided informed consent to participate in the Heart Health pilot study. If needed, research staff provided potential participants with telephone-based assistance in downloading the Lark smartphone app. We considered participants to have initiated the program after downloading the app, having their first conversation with the Lark AI coach, and completing basic onboarding and surveys (Day 5 in the program).

**Fig 1:**
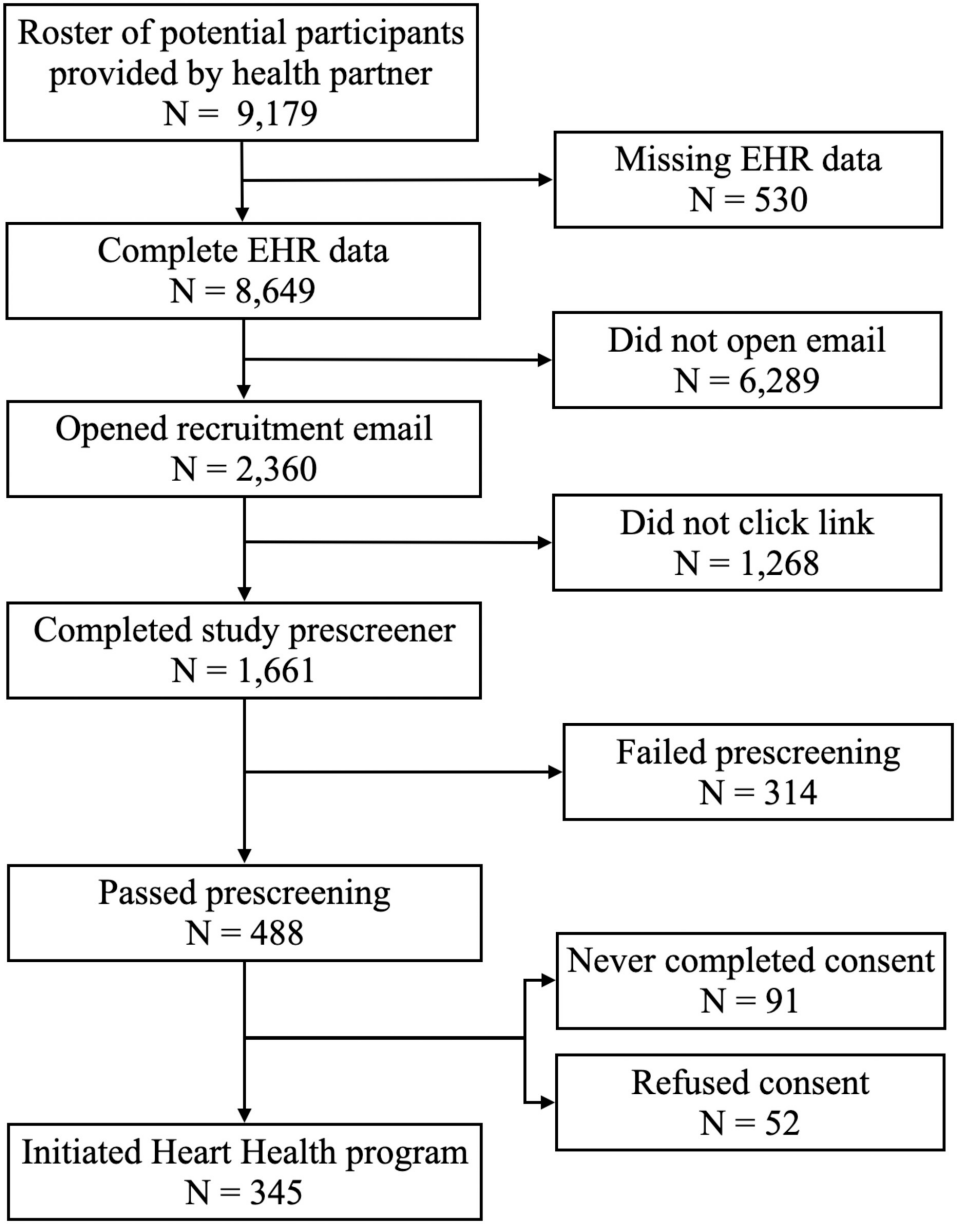
Flow chart for potential participants through the recruitment and enrollment process for the Heart Health study.

For flow of members through the recruitment and enrollment process, see Fig 1. Based on members with complete EHR data, the recruitment email open rate was 27.3% (2,360/8649) and the overall click through rate was 12.6% (1,092/8,649). Of members who opened the recruitment email, 46.3% (1,092/2,360) clicked on the personalized link and 73.4% (802/1,092) of those members submitted the prescreener.

### Data collection & measures

We used data from four sources for these analyses: (1) EHR data on participant demographics and health history, (2) recruitment data from email marketing campaigns through Lark’s healthcare partner (3) basic data from onboarding in the Lark Heart Health app, and (4) self-report screening surveys prior to or shortly after study initiation.

#### EHR measures

Lark’s healthcare partner, located in California, provided EHR data for potential participants on March 10, 2022, that included diagnostic history and recent medical record data from the year prior (March 10, 2021). All potential participants were individuals who opted in to receive wellness program information from their health provider and provided consent to share their data for research purposes. Table 1 provides descriptions of variables from the EHR. Health history variables were identified using the ICD-10 codes.

**Table 1:** Definitions and descriptive statistics for predictor variables from the EHR dataset.

Predictor variable (feature)	Operational Definition & ICD-10 code (if applicable)
Age	Age in years based on date of birth as of March 10, 2022
Sex	Biological sex (0 = female, 1 = male)
Race	Race/ethnic identity was divided into seven categories: White, Black, Hispanic/Latino, Asian American/Pacific Islander, Native American, Multiracial, no response
Body mass index (BMI)	BMI according to the following formula using most recently provided height and weight in the medical record: BMI = weight(kg)/height (m^2^)
Smoking status	Current smoking status (0 = not current smoker, 1 = current smoker)
Number of medications	Number of prescribed medications related to cardiometabolic risk.
Medication classes	Prescribed medications related to cardiometabolic risk were categorized by major cardiometabolic drug classes and split into six dichotomous medication variables:
	Statins
	Antihypertensive combination drugs
	Beta blockers
	Angiotensin converting enzyme (ACE) and angiotensin II inhibitors
	Insulin
	Antidiabetic combination drugs
Number of elevated lab values	Number of elevated lab values based on the latest report of the following and scored from 0–4:
	Low density lipoprotein (LDL) cholesterol ≥ 70 mg/dL
	Triglycerides ≥ 150 mg/dL
	Systolic blood pressure (SBP) ≥ 70 mm Hg
	HbA1c ≥ 6.5%
HbA1c	Percentage of glycated hemoglobin in the blood based on most recent report
Hypertension	History of essential hypertension (I10)
Atherosclerosis	History of atherosclerosis (I70.x)
Heart attack	History of heart attack (I21.x, I46.2, I46.9)
Stroke	History of stroke (I63.x, I69.x, G45.9, Z86.73)
Heart failure	History of heart failure (I50.x)
Heart surgery	History of heart surgery (I25.708, I25.709, I25.28, I25.729, I25.758, I97.2)
Aortic aneurysm and dissection	History of aortic aneurysm and dissection (I71.x)
Kidney failure/chronic kidney disease	History of kidney failure and chronic kidney disease (I12.0, N18.4, N18.5)
Diabetes status	Diabetes status was divided into three dichotomous variables:
	Type I diabetes (E10)
	Type II diabetes (E11)
	Other diabetes (E08, E09, E13)
Derived proxy ASCVD 10-year risk estimate	The derived proxy ASCVD 10-year risk estimate was divided into 5 categories based on the standard ASCVD 10-year risk categories:
	Low risk (<5%)
	Borderline risk (5% to 7.4%)
	Intermediate risk (7.5% to 19.9%)
	High risk (≥ 20%)
	Unscorable due to prior history of ASCVD or cardiac event

*Note*: ASCVD = atherosclerotic cardiovascular disease; HbA1c = glycated hemoglobin

A proxy ASCVD 10-year risk estimate was calculated using the following variables: current age, sex, race, systolic blood pressure, diastolic blood pressure, total cholesterol, high density lipoprotein cholesterol, history of diabetes, smoking status, and hypertension. This proxy score was calculated using published resources on the ASCVD Risk Assessment [26,27].

#### Outcome measures from recruitment campaign data and app

We assessed outcome measures based on data from the program’s email recruitment campaign. To test our primary objective, we used recruitment email *click through* as a proxy measure of program interest. For the purposes of these analyses, we defined click through as whether an individual clicked on their personalized link in the recruitment email for more information and to access the study prescreener. This metric should be considered a proxy measure of program interest because it assumes the following about the individual: (1) the email address was correct in the EHR database, (2) the email went to their inbox (i.e., did not go to spam), (3) they opened the email, and (4) they were interested enough in the recruitment email content to click the link for more information.

Additional outcome measures assessed included percentage of participants who opened the recruitment email, clicked on the personalized link in the recruitment email (i.e., clicked through), passed the prescreener, and initiated the Heart Health program. We operationalized program initiation as downloading the Heart Health app, having a first conversation with the Lark AI coach, and completing basic onboarding and surveys.

#### Participant characteristics measures from self-report screening surveys

We assessed participant characteristics for the study sample using a modified version of the “Non-Laboratory” Based INTERHEART Modifiable Risk Score survey [28,29]. To view the original instrument, see https://rome.phri.ca/interheartriskscore. This brief self-report questionnaire includes assessment of age, sex, height and weight (for calculation of body mass index), history of high blood pressure, history of diabetes, history of high blood pressure, parental history of heart attack, history of tobacco use and exposure, stress in the past year, depression in the past year, physical activity during leisure time, and typical dietary habits. We classified typical physical activity in leisure time as mainly sedentary, mild exercise with minimal effort, moderate exercise, or strenuous exercise. We classified typical dietary habits by assessing responses to five unhealthy diet items: 1) eating salty food daily, 2) eating deep fried foods 3x/week or more, and 3) eating meat 2x/day or more, 4) not eating fruit daily and 5) not eating vegetables daily. The screener also included added questions on whether the participant had a stroke or heart attack in the past.

### Statistical analysis and machine learning

#### Primary objective

We conducted all analyses using Python version 3.8.10. We used two different data-driven methods to evaluate EHR predictors of ***study interest*** based on click through on the personalized link in recruitment materials sent via email. Because of our data-driven approach, we did not have specific or directional hypotheses regarding which EHR variables would be key predictors; the only exception to this is that we anticipated that males would be less likely to show interest than females based on prior literature. The first method was a backward elimination regression assessing which EHR features predicted study interest. EHR features included demographic information, lab reading values, smoking status, prior clinical diagnoses, medications, and cardiac events described in Table 1. This stepwise regression model removed the least significant feature in the model until only significant features and required features (i.e., age, sex, race) remained in the model. This method enables assessment of the strongest predictors of interest and also provides insight into the directionality of relationships.

The second method used to address our primary objective was a machine learning technique called eXtreme Gradient Boost (XGB) [30]. We used the XGB model to determine the most impactful EHR variables for predicting study interest. XGB is an algorithm that trains a collection of gradient boosted decision trees to predict a target variable. In this case, the XGB model predicted the binary study interest outcome of “clicked” vs. “did not click.” The XGB model utilized the same EHR features as the backward elimination regression (Table 1). XGB is well-suited to tabular data problems with many correlated features, such as EHR data. During model training, we assessed model performance using the area under the receiver operating characteristic (AUC ROC) curve averaged over five cross-validation folds, with 10% of the data held out for testing. Reported performance metrics are weighted metrics to account for the impact of class imbalance. Important hyperparameters in the final model included use of a gbtree booster, a learning rate of 0.01, 240 trees, and a max depth of 6. Since XGB is a complex, nonlinear model, we used SHapley Additive exPlanations (SHAP) to understand the decisions made by the model [31]. Specifically, we used SHAP values to understand features contributing to why an individual was more or less likely to show interest in the study by clicking on the study link. The SHAP values quantify the contribution that each feature brings to the XGB model prediction (clicked/did not click). Summing the SHAP values of each feature of a given observation yields the difference between the prediction of the model and the null model. This method also is advantageous because it provides insight into potential nonlinear relationships between the EHR features and the binary outcome of study interest.

#### Secondary objectives

To assess Secondary Objective 1, we examined sex differences at key points in the program recruitment and initiation process. Specifically, we used Chi-square tests to determine whether there were significant sex differences in recruitment email open rates, recruitment email click through, prescreening success, and program initiation. To assess Secondary Objective 2, we tested for sex differences in key characteristics among those who initiated the Heart Health program using Chi-square tests and t-tests.

## Results

### EHR data descriptive statistics

Descriptive statistics for each of the EHR variables are shown in Table 2. On average, individuals in the EHR dataset were 66.1 years old (*median* = 68.8, *range* = 39.9–76.3) and approximately half of the individuals were male. The majority of the individuals identified as White (59.3%), with Hispanic/Latino individuals comprising 10% of the dataset, but nearly a quarter did not provide their race or ethnicity (24%). According to average BMI, individuals in the dataset were borderline obese (*M* = 30.3 kg/m^2^, *median* = 29.3 kg/m^2^, *range* = 18.0–59.8). Notably, almost two thirds of the individuals in the dataset had a diagnosis of hypertension and approximately 96% of individuals had at least one elevated lab value. More than half of the dataset (55%) were at intermediate or high risk for a cardiac event in the next 10 years, according to the proxy ASCVD 10-year risk score; another 27% of individuals could not be given an ASCVD score due to having a history of CVD, CAD, or a cardiac event. Over half (58%) of the individuals in the dataset were taking at least one medication. The most common prescribed medications for individuals in the EHR dataset were statins (46%) and antihypertensive combination drugs (25%).

**Table 2:** Descriptive statistics for EHR predictor variables.

	All Participants		Showed Interest (clicked through)		Did Not Show Interest (did not click through)	
	N = 8,649		n = 1,092		n = 7,557	
	N/n	Mean or %	n	Mean or %	n	Mean or %
Age (years)	8,649	66.1 (*SD* = 8.5)	1,092	65.8 (*SD* = 8.9)	7,557	66.1 (*SD =* 8.5)
Sex (% male)	4,260	49.3%	528	48.4%	3,732	49.4%
Race/ethnicity						
White	5,126	59.3%	689	63.0%	4,437	58.7%
Black	203	2.3%	22	2.0%	181	2.4%
Hispanic/Latino	867	10.0%	97	8.9%	770	10.2%
Asian American/Pacific Islander	251	2.9%	26	2.4%	225	3.0%
Native American	58	0.7%	8	0.7%	50	0.7%
Multiracial	47	0.5%	3	0.3%	44	0.6%
Not answered	2,097	24.2%	247	22.6%	1850	24.5%
Body mass index	8,649	30.3 (*SD* = 6.7)	1,092	30.8 (*SD* = 6.6)	7,557	30.2 (*SD =* 6.7)
Smoking status (% current smoker)	556	6.4%	40	3.7%	516	6.8%
Hypertension history	5,471	63.3%	681	62.4%	4,790	63.4%
Atherosclerosis history	2,005	23.2%	225	20.6%	1,780	23.6%
Heart attack history	9	0.0%	2	0.2%	7	0.1%
Stroke history	183	2.1%	20	1.8%	163	2.2%
Heart failure history	147	1.7%	22	2.0%	125	1.7%
Heart surgery history	15	0.2%	0	0.0%	15	0.2%
Aortic aneurysm/dissection history	42	0.5%	6	0.6%	36	0.5%
Type I diabetes history	59	0.7%	4	0.4%	55	0.7%
Type II diabetes history	2422	28.0%	270	24.7%	2,152	28.5%
Kidney disease/failure history	195	2.3%	16	1.5%	179	2.4%
Proxy ASCVD 10-year risk estimate	8,649	17.2 (*SD* = 12.5)	1,092	16.3 (*SD* = 11.8)	7,557	17.4 (*SD* = 12.5)
Proxy ASCVD category						
Low risk (<5%)	1,051	12.2%	156	14.3%	895	11.8%
Borderline risk (5–7.4%)	499	5.8%	68	6.2%	431	5.7%
Intermediate risk (7.5–19.9%)	2,575	29.8%	334	30.6%	2,241	29.7%
High risk (>20%)	2,178	25.2%	265	24.3%	1913	25.3%
Not scorable due to CVD history	2,346	27.1%	269	24.6%	2,077	27.5%
HbA1c	8,649	6.1 (*SD* = 1.1)	1,092	6.0 (*SD* = 1.0)	7,557	6.1 (*SD* = 1.1)
Number of elevated lab values						
0	313	3.6%	44	4.0%	269	3.6%
1	2,539	29.4%	358	32.8%	2,181	28.9%
2	3,640	42.1%	448	41.0%	3,192	42.2%
3	1,785	20.6%	211	19.3%	1,574	20.8%
4	372	4.3%	31	2.8%	341	4.5%
Number of medications						
0	3,629	42.0%	469	43.0%	3,160	41.8%
1	2,211	25.6%	283	25.9%	1,928	25.5%
2	1,493	17.3%	187	17.1%	1,306	17.3%
3	740	8.6%	84	7.7%	656	8.7%
4+	576	6.7%	69	6.3%	507	6.7%
Medication classes						
Statins	3,979	46.0%	514	47.1%	3,465	45.9%
Antihypertensive combination drugs	2,160	25.0%	254	23.3%	1,906	25.2%
Beta blockers	349	4.0%	44	4.0%	305	4.0%
ACE and angiotensin II inhibitors	1,080	12.5%	135	12.4%	945	12.5%
Insulin	361	4.2%	33	3.0%	328	4.3%
Antidiabetic combination drugs	684	7.9%	70	6.4%	614	8.1%

*Note*: ASCVD = atherosclerotic cardiovascular disease; CVD = cardiovascular disease; HbA1c = glycated hemoglobin; ACE = angiotensin converting enzyme

### Primary objective: Predicting interest with EHR data

The first method used to predict interest (i.e., click through) with EHR data was a backward regression model. The final model, including only significant and required predictors, is shown in Table 3. Results from the final model show the EHR features that predicted higher odds of study interest: higher BMI, fewer elevated lab values, and lower glycated hemoglobin (HbA1c). Individuals were also more likely to show interest if they were not current smokers or if they identified as White.

**Table 3:** Final model of backward elimination logit regression.

	*β*	*SE*	*z*	*p*
Constant	-1.96	0.03	-59.12	<.001
Sex (male)	-0.01	0.03	-0.37	.713
Race/ethnicity (White)	0.09	0.03	2.73	**.006**
Smoking Status (not current smoker)	0.16	0.04	3.84	**<.001**
Age	-0.03	0.03	-1.01	.313
Body mass index	0.12	0.03	3.54	**<.001**
HbA1c	-0.10	0.04	-2.41	**.016**
Number of elevated lab values	-0.10	0.04	-2.82	**.005**

*Note*: HbA1c = glycated hemoglobin

The majority of EHR predictors dropped out of the model due to nonsignificant relationships with interest. Nonsignificant relationships with program interest indicate that individuals had similar odds of clicking through at different levels of the predictor variable or that relationships could not be detected using linear regression methods. Individuals had similar odds of showing program interest regardless of sex, age, history of cardiovascular health issues, proxy ASCVD score, history of diabetes, history of chronic kidney disease or failure, number of medications, and use of different types of medications for CVD risk or diabetes.

**Fig 2:**
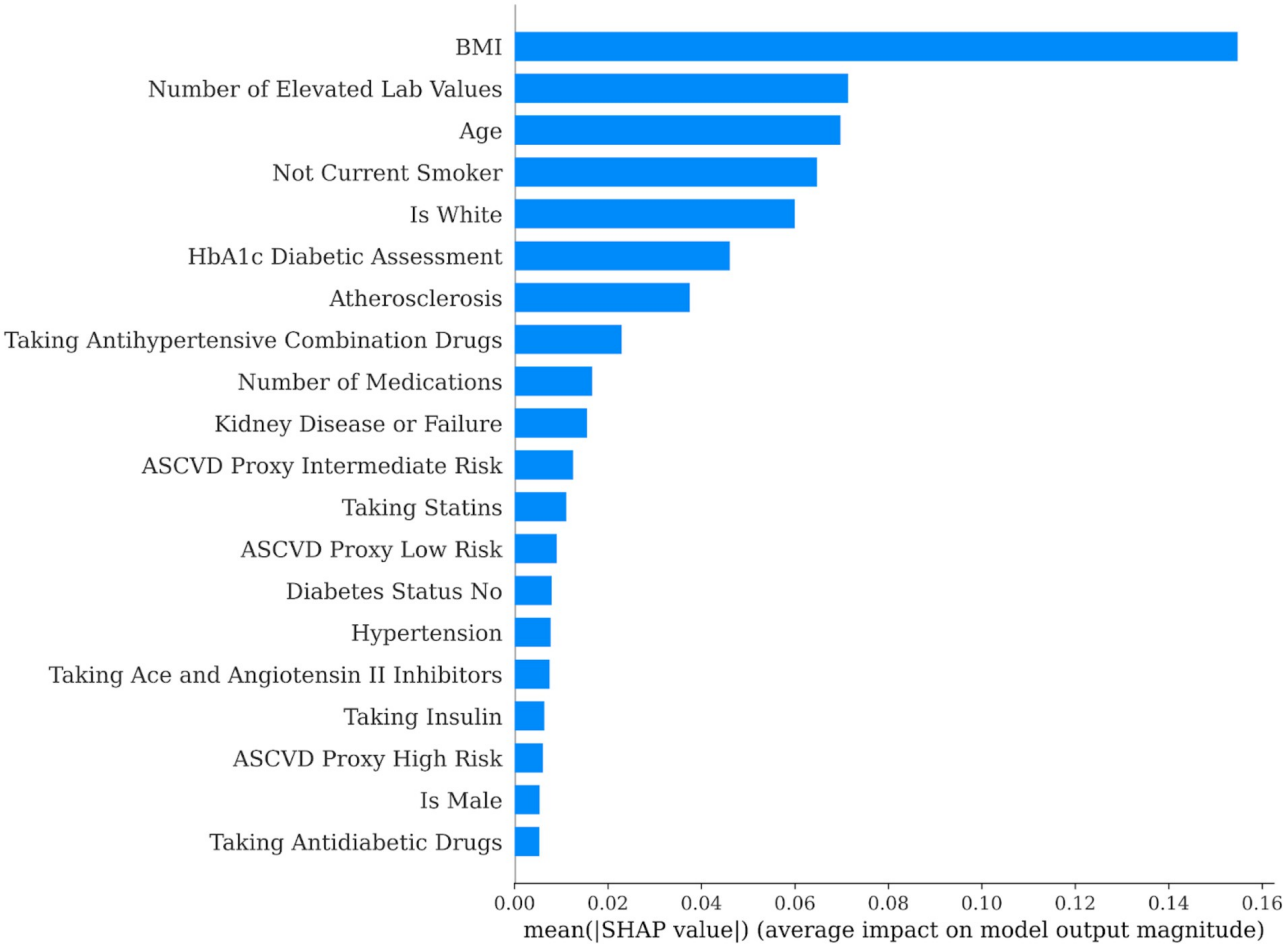
Forest plot showing the model features with the largest impact (i.e., highest SHAP values) in the XGB model predicting whether a potential participant showed interest (i.e., clicked through to find out more information after receiving the program recruitment email). The SHAP value represents the average marginal contribution of a feature across all possible combinations of features for a given observation.

**Fig 3:**
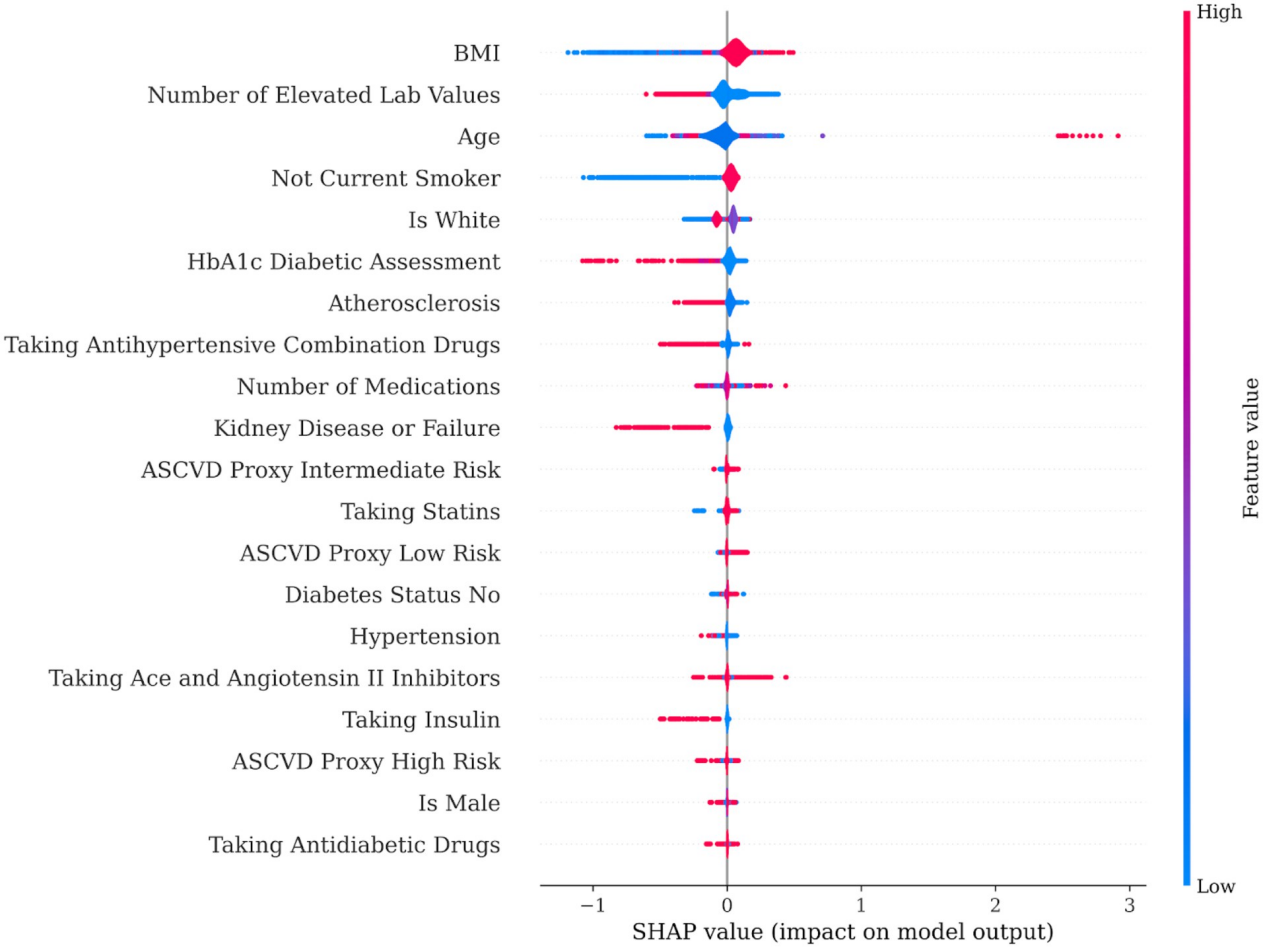
Violin plot showing SHAP values for features in the XGB model predicting whether a potential participant showed interest (i.e., clicked through to find out more information after receiving the program recruitment email). The SHAP value represents the average marginal contribution of a feature across all possible combinations of features for a given observation.

The second method used to predict interest (i.e., click through) with EHR data was an XGB machine learning approach. Overall, the model fit was poor with an average area under the curve of 0.56, accuracy of 42%, precision of 80%, recall of 34%, and an F1 score of 39%. These performance metrics indicate that this set of EHR features was not a strong predictor of program interest. For this reason, the impact level of individual features in the model should be interpreted with caution. As shown in Fig 2, the EHR features with the largest impact (i.e., highest SHAP values) when predicting interest were BMI, number of elevated lab values, age, smoking status, race, HbA1c, and history of atherosclerosis. Notably, all significant features from the regression model also showed high impact in the XGB model. Model features that did not have a high impact in the XGB model included sex, hypertension history, stroke history, heart attack history, heart failure history, heart surgery history, aortic aneurysm/dissection history, history of cardiovascular health issues, proxy ASCVD score, history of diabetes, history of chronic kidney disease or failure, number of medications, and use of different types of medications for CVD risk or diabetes. One advantage of XGB models is they can provide insights into potential nonlinear relationships that may be undetectable using regression methods. To visualize potential nonlinear relationships, Fig 3 shows a violin plot of SHAP values for each of the features. The SHAP value represents the average marginal contribution of a feature across all possible combinations of features for a given observation. In this plot, a nonlinear relationship would be signified by significant interspersion between high (red) and low (blue) feature values moving along the x-axis from low to high SHAP values. This is exemplified by the line for age in Fig 3, indicating that age had a nonlinear impact on program interest in these data.

### Secondary objectives: Examining sex differences

#### Secondary objective 1

To examine Secondary Objective 1, we first tested for sex differences at key points in the recruitment and program initiation process. As shown in Table 4, there were no sex differences at any point in the recruitment and study initiation funnel (all *p*’s >.05). That is, equal proportions of males and females opened the recruitment email, clicked on the personalized link in the recruitment email (i.e., clicked through), passed the prescreener, and initiated the Heart Health program.

**Table 4:** Sex differences at key points in the recruitment and study initiation process.

	Total N	Male		Female			
		n	%	n	%	Chi squared	p-value
Opened	2360	1178	49.9%	1182	50.1%	0.57	0.90
Did not open	6289	3082	49.0%	3207	51.0%		
Clicked	1092	528	48.4%	564	51.6%	1.99	0.57
Did not click	1268	650	51.3%	618	48.7%		
Passed prescreener	488	241	49.4%	247	50.6%	1.13	0.77
Failed prescreener	314	143	45.5%	171	54.5%		
Initiated study	345	162	47.0%	183	53.0%	0.16	0.68
Did not initiate study	143	70	49.0%	73	51.0%		

#### Secondary objective 2

To examine Secondary Objective 2, we tested for sex differences in baseline characteristics among participants who initiated the Heart Health program. Descriptive statistics for baseline characteristics are shown in Table 5. There were no sex differences in the majority of these variables, including age and BMI. Similarly, there were no sex differences in regard to race, type II diabetes history, tobacco use, and dietary habits. However, there were several baseline characteristics that differed by sex. Compared to females, a greater proportion of males had a history of high blood pressure (*p* = .015) and were using medication for high blood pressure (*p* = .015). Physical activity in leisure time also differed by sex (*p* <.001), with a greater proportion of females reporting that they were mainly sedentary and a greater proportion of males reporting that they regularly engaged in moderate exercise. There were also sex differences in depression history, with larger proportion of females reporting a history of depression over the past year compared to males (*p* <.001).

**Table 5:** Baseline characteristics of participants who the Heart Health Program.

	All Participants (N = 345)		Males (n = 162)		Females (n = 183)		*t* or chi-square	p-value
	n	Mean or %	n	Mean or %	n	Mean or %		
Age (years)		64.4 (*SD* = 9.0)		64.8 (*SD* = 8.4)		63.9 (*SD* = 9.1)	0.9	0.379
Sex (% male)	162	47%	162	100%	0	0%		
Race							5.3	.070
White	212	61.4%	100	61.7%	112	61.2%		
Black	15	4.3%	11	6.8%	4	2.2%		
Other	69	20.0%	28	17.3%	41	22.4%		
No response	49	14.2%	23	14.2%	26	14.2%		
Baseline BMI		32.2 (*SD* = 5.8)		31.7 (*SD* = 5.5)		32.7 (*SD* = 5.6)	1.6	0.106
High BP history	223	64.60%	116	71.6%	107	58.5%	5.9	**0.015**
High BP meds (prescreener)	212	86.20%	111	68.5%	101	55.2%	5.9	**0.015**
T2 diabetes history	75	21.70%	125	77.2%	145	79.2%	0.1	0.737
History of Depression	103	29.86%	31	19.1%	72	39.4%	15.8	**<.001**
Tobacco history (% never smokers)	326	94.50%	153	94.4%	173	94.5%	6.0	1.0
Physical activity in leisure time							18.6	**<.001**
Mainly sedentary	130	37.70%	48	29.6%	82	44.8%		
Mild exercise, low effort	138	40.00%	62	38.3%	76	41.5%		
Moderate exercise	77	22.30%	52	32.1%	25	13.7%		
Dietary habits								
Salty food (daily)	224	64.90%	100	61.7%	124	67.8%	1.1	.290
Fried food (3x/week or more)	107	31.00%	60	37.0%	47	25.7%	4.7	0.031
Meat (2x/day or more)	211	61.20%	104	64.2%	107	58.5%	1.0	0.33
Fruit (daily)	196	56.80%	89	54.9%	107	58.5%	0.3	0.581
Vegetables (daily)	275	79.70%	131	80.9%	144	78.7%	0.1	0.713

Notes: the chi square values for race and physical activity indicate the omnibus test statistic comparing across all categories of the respective variable

## Discussion

This is the first report from the ongoing Heart Health pilot study, in which we explored factors predicting interest in a digital health lifestyle change program for cardiovascular health. BMI, number of elevated lab values, HbA1c, smoking status, and race all emerged as key predictors of interest. Conversely, sex, age, history of CVD, history of diabetes, history of kidney issues, and medication use were not predictors of interest. This finding indicates that this fully digital Heart Health program may be equally appealing to both males and females, to adults across various age groups, and to individuals both with and without a history of cardiac and other chronic health issues. Taken together, these results contribute to the growing body of knowledge on the characteristics of those interested in digital health programs and provide insight into groups who could benefit from such programs but may need additional recruitment efforts.

### Predicting study interest

Using two data-driven methods, we explored a range of EHR variables that might predict interest in the Heart Health program. Although both methods identified similar relationships between the predictors and showing interest, the XGB method showed overall poor model performance; for this reason, we primarily focus our interpretation on the significant results from the regression model. Many of the factors proposed to play a large role in predicting interest in digital health programs (e.g., sex, age, disease diagnoses) [9,12] did not significantly predict interest in the regression model, indicating that these factors did not have a high impact on whether an individual showed interest in the program. These results support the long-term recruitment goals of the Heart Health program, which include appealing to individuals from a wide range of demographic groups who are at risk for CVD or in stable condition after a cardiac event.

There were several EHR variables that emerged as key predictors of study interest: BMI, number of elevated lab values, HbA1c, smoking status, and race. These variables were statistically significant in the final regression model and also showed high impact in the XGB model. We observed that individuals with higher BMI were more likely to show program interest. Given the elevated CVD risk associated with high BMI [32], those with higher BMI are a key group who could greatly benefit from this digital lifestyle coaching. Moreover, individuals with very high BMI tend to delay or avoid healthcare visits and tend to have lower participation in lifestyle change programs, in part due to emotional barriers, such as perceived weight stigma from providers [33]. We have previously shown that AI-driven lifestyle change programs tend to enroll higher rates of users with high BMI compared to in-person programs [34], suggesting that AI-driven coaching may be more appealing to individuals with higher BMIs, as it reduces the possibility of experiencing provider stigma. This is crucial, given that a key objective for the Heart Health program is reaching and engaging potential users with high BMI.

We also found that current smokers, individuals with a greater number of elevated lab values, and individuals with higher HbA1c were less likely to show interest. There are several potential explanations for these results, although they are largely speculative given the scarcity of evidence on digital health program interest: these individuals may 1) have already received educational materials or referrals focused for interventions; 2) be focusing primarily on pharmaceutical approaches; 3) be focused on another specific health issue (e.g., diabetes management) and unaware of the overlap with heart health; or 4) simply not be interested or ready to improve their health behaviors. Additional research is needed to explore these possible explanations. It is also notable that, although each of these variables were statistically significant predictors program interest, the magnitude of differences was quite small in the clinical context. For instance, HbA1c was 6.0% in individuals who showed interest versus 6.1% in those who did not show interest (Table 2). As such, we anticipate that these results will primarily be useful in the context of recruitment efforts and communications, rather than clinical decision making.

We also found that White individuals were more likely to show interest than those who did not identify as White or did not provide their race/ethnicity. However, this result should be interpreted cautiously due to limitations in racial/ethnic data and the program’s current availability to English speakers only. Notably, 24% of the individuals in the EHR dataset did not provide a racial/ethnic identity. A number of factors may contribute to a patient choosing not to provide their racial or ethnic identity to medical providers, such as privacy concerns and worries over bias from providers due to racial or ethnic identity [35,36]. As such, questions about race and ethnicity cannot be fully addressed with the present analyses but should be explored in future work, particularly given evidence for lower rates of digital health utilization for racial and ethnic minorities [13] and evidence of lower healthcare utilization and mistrust of medical providers and research among racial minority groups [36,37]. To promote health equity, digital health programs should prioritize inclusion and recruitment from diverse populations, considering cultural differences and language accessibility. Taken together, these results provide initial insights into groups of individuals who may be more or less interested in participating in a prevention-focused lifestyle change program for heart health, while also opening the door for expanding research focused on interest in digital health.

### Sex differences

Based on prior evidence that males are underrepresented in prevention-focused digital health programs and exhibit lower utilization of digital health tools [19–21], we anticipated that there would be greater proportions of females compared to males at key points in the recruitment and enrollment process. Contrary to our hypothesis, we found that males and females were equally represented at each point in the process. These results indicate that the Heart Health program may be more appealing to males than what might be expected from other prevention-focused digital health programs [19]. Although we cannot directly test the reason for the lack of sex differences in the present study, we can speculate on possible explanations. First, males tend to have greater risk of CVD at earlier ages compared to females [4,17]; this may lead to males having more awareness of their CVD risk, thus triggering greater interest in CVD-focused digital health programs compared to general prevention and wellness programs. Indeed, CVD awareness tends to be much lower among females compared to males, though awareness of CVD in females has increased in recent years [4,38]. Additionally, the Heart Health program may have attracted a greater proportion of males than expected because it was open to participants in both primary and secondary (stable) prevention stages. This would fit with findings from prior studies focused specifically on secondary CVD prevention in older adults which show that there are higher rates of men compared to women in digital health secondary prevention CVD interventions for older adults [39].

We also found sex differences among individuals who initiated the Heart Health program. Specifically, there was a higher proportion of males with a history of high blood pressure and medication use for high blood pressure, as well as a lower proportion of males reporting a history of depression. These sex differences are largely in line with epidemiological studies showing sex differences in these variables [1,40] although sex differences in medication use for high blood pressure differ by medication class [41–43].

### Strengths, limitations, and generalizability

There are several notable strengths to the present study. First, we were able to examine EHR data from a large sample from Lark’s health partner. Second, this EHR data enabled us to consider a wide range of possible predictors of study interest. Third, we utilized two data-driven approaches to explore these predictors of study interest, enabling us to compare results across multiple methods. Finally, exclusion criteria for the Heart Health pilot study were not as stringent as clinical trials, so analytics on the recruitment process for this program are closer to “real world” enrollment in a digital health program than a clinical trial.

There are also several limitations to the analyses. First, a single healthcare partner in California provided the potential study sample and the sample primarily included older adults. As such, these results might not generalize to other regions of the United States or younger samples. Given that we trained these models on a single dataset and did not externally validate them with a separate test set using a different patient population, it is possible that features of importance in this model would not consistently emerge or generalize to new and different patient populations. Important next steps in this line of work include exploring the utility of the models with EHR data from other healthcare partners who have different patient populations. Drawing from a single healthcare partner also limits characteristics of the study population, such as race and ethnicity or other sociodemographic factors. These factors should all be considered as limitations to generalizability of the present results. Second, we took a data-driven, exploratory approach to test the impact of a wide variety of predictors and the results should be interpreted as such. Third, we focused on “click through” as a proxy measure for study interest, but this measure does have several caveats mentioned in the methods section. Lastly, these analyses are specific to the Lark Heart Health program; as a result, the present findings do not necessarily generalize to other digital health offerings and are not generalizable to in-person or telehealth programs.

### Future directions

Although these analyses are specific to the Lark Heart Health pilot study, there are several future directions from these results that can help inform other digital health programs. First, exploring factors related to response variables such as recruitment email click through can provide insight into how to tailor the program to those who are already interested (e.g., users with higher BMI). Second, this work can help spur ideas for increasing interest among those who could benefit from digital health programs but do not show high initial interest. For example, perhaps individuals with more elevated lab values would show greater interest in the program if lowering their lab values was related to some type of incentive from the program or from their provider. Third, these analyses highlight the need to develop strategies to “activate” individuals who could benefit from lifestyle change programs but do not feel ready to take action toward improving their health. An important next step in this line of work is to examine additional factors that predict program interest and participation that could not be found in EHR data. For instance, previous work has identified variables that predict lack of program participation and utilization of digital health tools, including insufficient technical and digital literacy skills, lack of interest in digital health, preference for in-person treatment, and lack of time [12,23]. Future work could also examine how these factors interact with EHR predictors of study interest. Additionally, study interest should be examined in broad and diverse samples across different regions, racial/ethnic groups, health backgrounds, age groups, and socioeconomic groups to expand the generalizability of the findings and reduce potential sample-related bias. Finally, given the caveats of using “click through” as a measure of study interest, future studies should explore other measures of study interest beyond click through.

## Conclusions

In conclusion, our findings contribute to understanding the characteristics of individuals interested in a prevention-focused, digital health program for heart health. We found higher interest among individuals with higher BMI, those with fewer elevated lab values and lower A1c, nonsmokers, and White individuals. Additionally, we found that there was no significant difference in program interest based on sex, age, history of cardiovascular and other chronic health issues, proxy ASCVD score, number of medications, and use of different types of medications for CVD risk or diabetes. Moreover, we found no sex differences in the recruitment and enrollment process. These insights can aid in refining the Heart Health program to best serve those who are interested in participating, as well as help to highlight groups of individuals who may benefit from such a program but need additional recruitment efforts or tailoring of the program to increase their interest. These results add to the growing literature focused on making digital health lifestyle change programs accessible and valuable to all individuals who can benefit from lifestyle change programs for chronic disease prevention.

## References

[1] Tsao CW, Aday AW, Almarzooq ZI, Alonso A, Beaton AZ, Bittencourt MS, et al. Heart Disease and Stroke Statistics—2022 Update: A Report From the American Heart Association. Circulation. 2022;145(8):e153–639. doi: 10.1161/CIR.0000000000001052 35078371

[2] Benjamin EJ, Virani SS, Callaway CW, Chamberlain AM, Chang AR, Cheng S, et al. Heart Disease and Stroke Statistics—2018 Update. Circulation. 2018;137(12):e67–492. 29386200 10.1161/CIR.0000000000000558

[3] Arnett DK, Blumenthal RS, Albert MA, Buroker AB, Goldberger ZD, Hahn EJ, et al. 2019 ACC/AHA Guideline on the Primary Prevention of Cardiovascular Disease: A Report of the American College of Cardiology/American Heart Association Task Force on Clinical Practice Guidelines. Circulation [Internet]. 2019 Sep 10 [cited 2023 Feb 1];140(11). Available from: https://www.ahajournals.org/doi/10.1161/CIR.0000000000000678 10.1161/CIR.0000000000000678 PMC7734661 30879355

[4] Peters SAE, Muntner P, Woodward M. Sex Differences in the Prevalence of, and Trends in, Cardiovascular Risk Factors, Treatment, and Control in the United States, 2001 to 2016. Circulation. 2019;139(8):1025–35. doi: 10.1161/CIRCULATIONAHA.118.035550 30779652

[5] Krist AH, Davidson KW, Mangione CM, Barry MJ, Cabana M, Caughey AB, et al. Behavioral Counseling Interventions to Promote a Healthy Diet and Physical Activity for Cardiovascular Disease Prevention in Adults With Cardiovascular Risk Factors. JAMA. 2020;324(20):2069–75. 33231670 10.1001/jama.2020.21749

[6] Mangione CM, Barry MJ, Nicholson WK, Cabana M, Coker TR, Davidson KW, et al. Behavioral Counseling Interventions to Promote a Healthy Diet and Physical Activity for Cardiovascular Disease Prevention in Adults Without Cardiovascular Disease Risk Factors. JAMA. 2022;328(4):367–74. 35881115 10.1001/jama.2022.10951

[7] Patnode CD, Redmond N, Iacocca MO, Henninger M. Behavioral Counseling Interventions to Promote a Healthy Diet and Physical Activity for Cardiovascular Disease Prevention in Adults Without Known Cardiovascular Disease Risk Factors. JAMA. 2022;328(4):375–88. 35881116 10.1001/jama.2022.7408 PMC13326174

[8] Kuan PX, Chan WK, Ying DKF, Rahman MAA, Peariasamy KM, Lai NM, et al. Efficacy of telemedicine for the management of cardiovascular disease: a systematic review and meta-analysis. Lancet Digit Health. 2022;4(9):e676–91. doi: 10.1016/S2589-7500(22)00124-8 36028290 PMC9398212

[9] Mahajan S, Lu Y, Spatz ES, Nasir K, Krumholz HM. Trends and Predictors of Use of Digital Health Technology in the United States. Am J Med. 2020;134(1):129–34. doi: 10.1016/j.amjmed.2020.06.033 32717188

[10] Frederix I, Caiani EG, Dendale P, Anker S, Bax J, Böhm A, et al. ESC e-Cardiology Working Group Position Paper: Overcoming challenges in digital health implementation in cardiovascular medicine. Eur J Prev Cardiol. 2019;26(11):1166–77. doi: 10.1177/2047487319832394 30917695

[11] Bhardwaj V, Spaulding EM, Marvel FA, LaFave S, Yu J, Mota D, et al. Cost-effectiveness of a Digital Health Intervention for Acute Myocardial Infarction Recovery. Med Care. 2021;59(11):1023–30. doi: 10.1097/MLR.0000000000001636 34534188 PMC8516712

[12] Brouwers RWM, Brini A, Kuijpers RWFH, Kraal JJ, Kemps HMC. Predictors of non-participation in a cardiac telerehabilitation programme: a prospective analysis. Eur Heart J—Digit Health. 2021;3(1):ztab105-. 10.1093/ehjdh/ztab105 PMC9707959 36713984

[13] Nouri SS, Adler-Milstein J, Thao C, Acharya P, Barr-Walker J, Sarkar U, et al. Patient characteristics associated with objective measures of digital health tool use in the United States: A literature review. J Am Med Inform Assoc. 2020;27(5):834–41. doi: 10.1093/jamia/ocaa024 32364238 PMC7309253

[14] Evangelista L, Steinhubl SR, Topol EJ. Digital health care for older adults. The Lancet. 2019;393(10180):1493. doi: 10.1016/S0140-6736(19)30800-1 30983579 PMC8106920

[15] Auster-Gussman LA, Lockwood KG, Graham SA, Pitter V, Branch OH. Engagement in Digital Health App-Based Prevention Programs Is Associated With Weight Loss Among Adults Age 65+. Front Digit Health. 2022;4:886783. doi: 10.3389/fdgth.2022.886783 35663278 PMC9160365

[16] Graham SA, Stein N, Shemaj F, Branch OH, Paruthi J, Kanick SC. Older adults engage with personalized digital coaching programs at rates that exceed those of younger adults. Front Digit Health. 2021;3:642818. doi: 10.3389/fdgth.2021.642818 34713112 PMC8521864

[17] Leening MJG, Ferket BS, Steyerberg EW, Kavousi M, Deckers JW, Nieboer D, et al. Sex differences in lifetime risk and first manifestation of cardiovascular disease: prospective population based cohort study. BMJ. 2014;349(nov17 9):g5992. doi: 10.1136/bmj.g5992 25403476 PMC4233917

[18] Pinkhasov RM, Wong J, Kashanian J, Lee M, Samadi DB, Pinkhasov MM, et al. Are men shortchanged on health? Perspective on health care utilization and health risk behavior in men and women in the United States. Int J Clin Pract. 2010;64(4):475–87. doi: 10.1111/j.1742-1241.2009.02290.x 20456194

[19] Paradis S, Roussel J, Bosson JL, Kern JB. Use of Smartphone Health Apps Among Patients Aged 18 to 69 Years in Primary Care: Population-Based Cross-sectional Survey. JMIR Form Res. 2022;6(6):e34882. doi: 10.2196/34882 35708744 PMC9247815

[20] Antezana G, Venning A, Smith D, Bidargaddi N. Do young men and women differ in well-being apps usage? Findings from a randomised trial. Health Informatics J. 2022;28(1):146045822110648. doi: 10.1177/14604582211064825 35128952

[21] Bol N, Helberger N, Weert JCM. Differences in mobile health app use: A source of new digital inequalities? Inf Soc. 2018;34(3):183–93.

[22] Escoffery C. Gender Similarities and Differences for e-Health Behaviors Among U.S. Adults. Telemed E-Health. 2018;24(5):335–43. doi: 10.1089/tmj.2017.0136 28813630

[23] Cheng C, Gearon E, Hawkins M, McPhee C, Hanna L, Batterham R, et al. Digital Health Literacy as a Predictor of Awareness, Engagement, and Use of a National Web-Based Personal Health Record: Population-Based Survey Study. J Med Internet Res. 2022;24(9):e35772. doi: 10.2196/35772 36112404 PMC9526109

[24] Branch OH, Rikhy M, Auster-Gussman LA, Lockwood KG, Graham SA. Relationships Between Blood Pressure Reduction, Weight Loss, and Engagement in a Digital App–Based Hypertension Care Program: Observational Study. JMIR Form Res. 2022;6(10):e38215. doi: 10.2196/38215 36301618 PMC9650575

[25] Graham SA, Pitter V, Hori JH, Stein N, Branch OH. Weight loss in a digital app-based diabetes prevention program powered by artificial intelligence. Digit Health. 2022;8:20552076221130620. 10.1177/20552076221130619 PMC9551332 36238752

[26] Goff DC, Lloyd-Jones DM, Bennett G, Coady S, D’Agostino RB, Gibbons R, et al. 2013 ACC/AHA Guideline on the Assessment of Cardiovascular Risk A Report of the American College of Cardiology/American Heart Association Task Force on Practice Guidelines. J Am Coll Cardiol. 2014;63(25):2935–59. 24239921 10.1016/j.jacc.2013.11.005 PMC4700825

[27] Lloyd-Jones DM, Huffman MD, Karmali KN, Sanghavi DM, Wright JS, Pelser C, et al. Estimating Longitudinal Risks and Benefits From Cardiovascular Preventive Therapies Among Medicare Patients The Million Hearts Longitudinal ASCVD Risk Assessment Tool: A Special Report From the American Heart Association and American College of Cardiology. J Am Coll Cardiol. 2017;69(12):1617–36. doi: 10.1016/j.jacc.2016.10.018 27825770 PMC5370170

[28] McGorrian C, Yusuf S, Islam S, Jung H, Rangarajan S, Avezum A, et al. Estimating modifiable coronary heart disease risk in multiple regions of the world: the INTERHEART Modifiable Risk Score. Eur Heart J. 2011;32(5):581–9. doi: 10.1093/eurheartj/ehq448 21177699

[29] Yusuf S, Rangarajan S, Teo K, Islam S, Li W, Liu L, et al. Cardiovascular Risk and Events in 17 Low-, Middle-, and High-Income Countries. N Engl J Med. 2014;371(9):818–27. doi: 10.1056/NEJMoa1311890 25162888

[30] Sheridan RP, Wang WM, Liaw A, Ma J, Gifford EM. Extreme Gradient Boosting as a Method for Quantitative Structure–Activity Relationships. J Chem Inf Model. 2016;56(12):2353–60. doi: 10.1021/acs.jcim.6b00591 27958738

[31] Lundberg S, Lee SI. A Unified Approach to Interpreting Model Predictions. arXiv. 2017;

[32] Kinlen D, Cody D, O’Shea D. Complications of obesity. QJM Int J Med. 2017;111(7):437–43. 10.1093/qjmed/hcx152 29025162

[33] Alberga AS, Edache IY, Forhan M, Russell-Mayhew S. Weight bias and health care utilization: a scoping review. Prim Health Care Res Dev. 2019;20:e116. doi: 10.1017/S1463423619000227 32800008 PMC6650789

[34] Auster-Gussman LA, Lockwood KG, Graham SA, Stein N, Branch OH. Reach of a Fully Digital Diabetes Prevention Program in Health Professional Shortage Areas. Popul Health Manag. 2022; 10.1089/pop.2021.0283 PMC9419962 35200043

[35] Hall WJ, Chapman MV, Lee KM, Merino YM, Thomas TW, Payne BK, et al. Implicit Racial/Ethnic Bias Among Health Care Professionals and Its Influence on Health Care Outcomes: A Systematic Review. Am J Public Health. 2015;105(12):e60–76. doi: 10.2105/AJPH.2015.302903 26469668 PMC4638275

[36] Vela MB, Erondu AI, Smith NA, Peek ME, Woodruff JN, Chin MH. Eliminating Explicit and Implicit Biases in Health Care: Evidence and Research Needs. Annu Rev Public Health. 2022;43(1):1–25. doi: 10.1146/annurev-publhealth-052620-103528 35020445 PMC9172268

[37] Bazargan M, Cobb S, Assari S. Discrimination and Medical Mistrust in a Racially and Ethnically Diverse Sample of California Adults. Ann Fam Med. 2021;19(1):4–15. doi: 10.1370/afm.2632 33431385 PMC7800756

[38] Mosca L, Mochari-Greenberger H, Dolor RJ, Newby LK, Robb KJ. Twelve-Year Follow-Up of American Women’s Awareness of Cardiovascular Disease Risk and Barriers to Heart Health. Circ Cardiovasc Qual Outcomes. 2010;3(2):120–7. doi: 10.1161/CIRCOUTCOMES.109.915538 20147489 PMC2956447

[39] Schorr EN, Gepner AD, Dolansky MA, Forman DE, Park LG, Petersen KS, et al. Harnessing Mobile Health Technology for Secondary Cardiovascular Disease Prevention in Older Adults: A Scientific Statement From the American Heart Association. Circ Cardiovasc Qual Outcomes. 2021;14(5):e000103. doi: 10.1161/HCQ.0000000000000103 33793309

[40] Lim GY, Tam WW, Lu Y, Ho CS, Zhang MW, Ho RC. Prevalence of Depression in the Community from 30 Countries between 1994 and 2014. Sci Rep [Internet]. 2018 Feb 12 [cited 2023 May 10];8(1):2861. Available from: https://www.nature.com/articles/s41598-018-21243-x 29434331 10.1038/s41598-018-21243-x PMC5809481

[41] Osude N, Durazo-Arvizu R, Markossian T, Liu K, Michos ED, Rakotz M, et al. Age and sex disparities in hypertension control: The multi-ethnic study of atherosclerosis (MESA). Am J Prev Cardiol [Internet]. 2021 Aug 8 [cited 2023 May 10];8:100230. Available from: https://www.ncbi.nlm.nih.gov/pmc/articles/PMC8367853/ 34430952 10.1016/j.ajpc.2021.100230 PMC8367853

[42] Wang J, Jiang W, Sharma M, Wu Y, Li J, You N, et al. Sex differences in antihypertensive drug use and blood pressure control. Postgrad Med J [Internet]. 2019 Jun 1 [cited 2023 May 10];95(1124):295–9. Available from: https://pmj.bmj.com/content/95/1124/295 31171709 10.1136/postgradmedj-2019-136513

[43] Zhao M, Woodward M, Vaartjes I, Millett ERC, Klipstein-Grobusch K, Hyun K, et al. Sex Differences in Cardiovascular Medication Prescription in Primary Care: A Systematic Review and Meta-Analysis. J Am Heart Assoc Cardiovasc Cerebrovasc Dis [Internet]. 2020 Jun 20 [cited 2023 May 10];9(11):e014742. Available from: https://www.ncbi.nlm.nih.gov/pmc/articles/PMC7429003/10.1161/JAHA.119.014742 PMC7429003 32431190

